# The Hidden Majority: Dual-Factor Mental Health Profiles Among Help-Avoidant University Students

**DOI:** 10.1007/s10964-026-02380-3

**Published:** 2026-07-03

**Authors:** Yuhan Hu, Marloes Nederhand, Ruth Van der Hallen, Pauline W. Jansen

**Affiliations:** Department of Psychology, Education & Child Studies, Erasmus University Rotterdam, Rotterdam, 3062 PA The Netherlands

**Keywords:** Dual-factor mental health, Help avoidance, Well-being, Profiles

## Abstract

Many university students experience mental health difficulties, yet a large proportion do not seek professional psychological support. Although prior research has linked mental health status to help-seeking behavior, it remains unclear whether all help-avoidant students require professional support and how their mental health profiles compare with those of help-seeking students. To address this gap, this study employed a dual-factor model, which integrates subjective well-being and psychopathology to map mental health profiles, and explored demographic predictors of profile membership. Additionally, profiles of help-avoidant students were also compared with those of students currently seeking help or who had sought help previously. The sample included 8,679 university students from seven Dutch universities (*M*_age_ = 21.76, *SD* = 3.31; 69.3% female), including 2,023 students who experienced mental health difficulties but did not seek help (*M*_age_ = 21.43, *SD* = 3.21; 71.7% female). Most of these help-avoidant participants were bachelor’s students (69.1%), freshmen (55.4%), and of Dutch origin (53.8%). Results supported the dual-factor model, identifying four distinct profiles: (1) “Complete mental health” (31.0%), characterized by high well-being and low psychopathology; (2) “Symptomatic but Content” (23.7%), with high-average psychopathology and relatively high well-being; (3) “Vulnerable” (27.7%), with low-to-average well-being and moderately low psychopathology; and (4) “Troubled” (17.6%), with low well-being and high psychopathology. Profile structures among help-avoidant students differed from those of current and past help-seeking group. Students in the “Complete Mental Health” Profile were more likely to be younger, female, domestic, master’s students, and non-freshman. Furthermore, insomnia severity increased progressively from the “Complete Mental Health” profile to the “Troubled” profile. These findings highlight the heterogeneity of mental health profiles among help-avoidant students, underscoring the importance of comprehensive screening, which may provide insights for targeted intervention strategies.

**Supplementary Information:**

The online version contains supplementary material available at 10.1007/s10964-026-02380-3.

## Introduction

University students face increasing mental health challenges. Approximately 25% report clinically significant depressive symptoms (Sheldon et al., 2021), 39% experience anxiety symptoms (Li et al., 2022), and 11% report severe stress (Beiter et al., 2015) during their studies. In addition, nearly half of students report persistent and problematic procrastination (Steel, 2007). Likewise, a large-scale survey conducted in the United Kingdom revealed a similar trend: the 2020 National Student Survey revealed that 42% of students exhibited mental health symptoms warranting professional support (Pereira et al., 2020). However, despite growing awareness and accessible resources, only one third of students reporting mental health problems had sought help from counseling services (Lui et al., 2024). Help-avoidant students, in particular, appear to face more chronic stressors and express greater concerns about professional help (Savage et al., 2016), highlighting the need of identifying and addressing help-avoidance tendencies as a complementary strategy to support psychological services. However, the mental health status of this subgroup remains poorly understood. It is unclear whether help-avoidant students indeed require professional intervention or how severe their mental health difficulties may be, particularly when considering the combined roles of well-being and psychopathology in shaping overall mental functioning. To address this gap, this study used latent profile analyses (LPA) grounded in the dual-factor model, which integrates subjective well-being and psychopathology to capture a more complete picture of mental health among help-avoidant students.

### Students Who Avoid Professional Help

Professional help-avoidance refers to the reluctance or refusal to seek available professional mental health support, despite the experience of mental health problems. It can be expressed behaviorally through avoidance of professional services and cognitively through a disconnect between experienced psychological distress and help-seeking intentions (Wilson & Deane, 2010). Importantly, help-avoidance is generally considered to reflect a motivated decision (Täuber, 2017). Therefore, help-avoidant students differ from individuals who do not seek help because they either do not perceive a need for treatment or face limited access to services.

Investigating the mental health status of help-avoidant students is particularly important, as untreated mental health difficulties have been associated with more severe psychopathology, including depression (Yamaguchi et al., 2023) and anxiety (Cheung et al., 2017). Moreover, reliance on avoidant coping strategies may contribute to poorer well-being later in life (Jiang et al., 2025). At the same time, some evidence suggests that a small proportion of help-avoidant students are able to resolve their difficulties without professional support (Savage et al., 2016). These findings indicate that help-avoidant students are likely to represent a heterogeneous group. While some students may experience relatively mild distress that resolves without formal treatment, others may face more serious mental health problems that remain unaddressed. Understanding this heterogeneity is important for improving early identification, informing university mental health services, and guiding targeted prevention and intervention efforts for students who may genuinely require support.

While structural barriers (e.g., financial cost, service accessibility) may prevent individuals from accessing care even when they intend to seek help, help-avoidance is more often driven by attitudinal barriers (Gulliver et al., 2010). These barriers reflect internal beliefs, preferences, and perceptions that reduce motivation to seek or continue treatment, including negative expectations about treatment outcomes and beliefs that symptoms will resolve without professional intervention (Andrade et al., 2014). Evidence consistently shows that attitudinal barriers are more prevalent than structural barriers. In particular, stigma and embarrassment, limited mental health literacy (i.e., difficulty recognizing symptoms and the need for care; Gulliver et al., 2010), and a preference for self-reliance (Ebert et al., 2019) are among the most frequently reported impediments to help-seeking among young people.

Beyond these barriers, mental health status itself may also be related to help avoidance (Russell et al., 2008). Some studies indicate that higher levels of anxiety (Fine et al., 2018) and perceived stress (Zochil & Thorsteinsson, 2018) are associated with greater likelihood of seeking professional support. Similarly, greater psychological distress and impaired psychosocial functioning have been linked to increased help-seeking intentions (Vogel & Wei, 2005). However, other findings suggest the opposite pattern. Students in “at-risk” or “low” well-being groups have been found to report significantly lower intentions to seek professional help compared to those in the “normal” well-being group (McCabe et al., 2024). Likewise, higher levels of depressive symptoms have been associated with lower subsequent help-seeking (Yamaguchi et al., 2023), which may be due to reduced motivation, feelings of hopelessness, and diminished expectations of support. In addition, certain behavioral tendencies may also relate to help avoidance. For instance, higher levels of procrastination have been linked to reduced engagement in mental health help-seeking behaviors (Stead et al., 2010). Taken together, these mixed findings suggest that different combinations of well-being and psychopathology may shape patterns of help seeking and avoidance. However, most prior studies have relied on variable-centered approaches, which disregards the complexity of mental health. Latent mental health profiles may provide more insight into the group of help-avoidant students, particularly if based on both psychopathological symptoms and well-being.

Importantly, it also remains unclear whether the latent structure of mental health differs across help-seeking groups. Latent profile analysis conducted in a U.S. sample of Asian Indian adults identified three mental health profiles: (1) low symptom severity, (2) high internalizing symptoms, and (3) high-risk symptoms. Internal barriers to help-seeking were highest among those who reported elevated but not extreme symptoms (Jin et al., 2023). However, it remains unclear whether help-avoidant students constitute a uniquely patterned subgroup or simply represent different levels of symptom severity relative to help-seeking students. Emerging evidence suggests that help-seeking behavior may vary not only by symptom severity but also by the pattern and type of symptoms, pointing to potential differences in underlying mental health profiles. For example, individuals with anxiety disorders are less likely to pursue professional help than those with unipolar depression (Johnson & Coles, 2013). Moreover, heterogeneity likely exists within help-seeking groups. Prior research shows that students currently receiving support report the highest levels of distress, whereas those with past help-seeking experience report somewhat lower symptom levels, though still elevated compared to help-avoidant students (Broglia et al., 2021). Similarly, individuals with a history of help-seeking report higher psychological symptoms than those who avoid services (Filiatreau et al., 2021). Together, these findings suggest that help-seeking status may reflect qualitatively different patterns of mental health rather than differences in severity alone. Understanding these distinctions may advance theoretical understanding of help-seeking behavior and clarify whether tailored interventions are warranted for help-avoidant students.

### Conceptualizing Complete Mental Health

Historically, due to psychology’s roots in the medical model of health and illness, mental health and mental illness were conceptualized as opposite ends of a single continuum, with mental health defined merely as the absence of psychopathology (Greenspoon & Saklofske, 2001). However, research shows that well-being and psychopathology are related yet distinct, and individuals can experience high well-being despite the presence of mental illness symptoms (Keyes, 2005). Specifically, mental health is conceived as a complete state in which individuals are free of psychopathology and flourishing with high levels of emotional, psychological, and social well-being (Keyes, 2005).

**Fig. 1:**
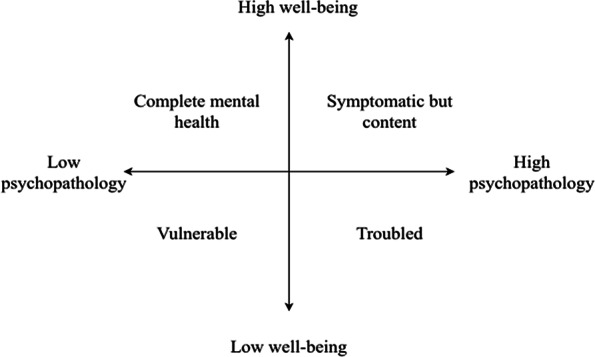
Dual-Factor Model of Mental Health. Note. The horizontal axis represents psychopathology from low to high, and the vertical axis represents well-being from low to high.

The dual-factor model of mental health classifies individuals into four distinct mental health groups (see Fig. 1): (a) “Complete Mental Health”—high well-being and low psychopathology; (b) “Symptomatic but Content”—high well-being despite high psychopathology; (c) “Vulnerable”—low well-being with low psychopathology and (d) “Troubled”—low well-being and high psychopathology (Suldo & Shaffer, 2008). Reviews have shown that empirical studies consistently support the utility of this model in predicting both psychological and behavioral outcomes (Iasiello et al., 2020). For instance, adolescents with “Troubled” (low well-being, high psychopathology), “Symptomatic but Content” (high well-being, high psychopathology), and “Vulnerable” (low well-being, low psychopathology) profiles were significantly more likely to engage in health-risk behaviors, such as cigarette smoking, alcohol consumption, low physical activity, and insufficient sleep, compared with those who exhibited complete mental health (high well-being, low psychopathology; Venning et al., 2013).

Although so far no studies have directly investigated the dual-factor mental health profiles of help-avoidant students, this framework has been applied in other contexts (Venning et al., 2013). Most prior research has relied on cut points to identify and categorize individuals with high versus low levels of well-being and psychopathology into four mental health groups (e.g., Greenspoon & Saklofske, 2001). However, this binary approach may oversimplify complex mental health patterns and an empirically driven person-centered approach may be more advantageous (Moore et al., 2019). Latent profile analysis is a model-based method that identifies unobserved subgroups based on individuals’ response patterns to continuous indicators. Unlike traditional cluster analysis, LPA uses probability-based classification and fit indices to determine the optimal number of profiles, increasing rigor and replicability (Herman et al., 2007).

Using LPA, previous studies have partially or fully supported the dual-factor model. For example, all four mental health profiles consistent with the dual-factor framework have been identified among secondary school students in Grades 9–11 (Moore et al., 2019). The dual-factor model has also been applied in higher education. Among college freshmen, four profiles were identified: “Flourishing Mental Health,” “Moderate Mental Health,” “Content-dominated but Symptomatic,” and “Symptom-dominated but Content”, which were partially consistent with the dual-factor model. Moreover, multinomial logistic regression analyses revealed that other variables can impact profile membership. For example, higher levels of both proactive personality and perceived university environment (reflecting how welcomed and comfortable students feel in their educational setting) predicting greater likelihood of belonging to the flourishing profile rather than suboptimal profiles (Chen et al., 2025). Similarly, LPA conducted among a large sample of Chinese university students supported a three-profile solution comprising “Flourishing”, “Vulnerable”, and “Troubled” groups, with the anticipated fourth “Symptomatic but Content” profile not emerging. Further analyses showed that academic emotions, defined as emotions directly linked to academic learning, classroom instruction, and achievement, also differentiated profile membership. Positive high- and low-arousal emotions increased the likelihood of belonging to the “Flourishing” group, whereas negative high-arousal emotions predicted membership in the “Troubled” group (Jiang et al., 2023). Collectively, these studies demonstrate the dual-factor model’s utility in educational contexts. However, these previous studies do not show whether the model applies to other groups, such as students who avoid professional help.

### Characteristic Related to Mental Health Group

Demographic factors are important correlates of students’ mental health profiles. For example, sex has been found to significantly predict mental health profile membership among adolescents (Ma & Lai, 2018). Compared with females, males were more likely to belong to the less mentally healthy classes. This pattern has also been observed in dual-factor study, with girls being more likely than boys to belong to “Troubled” or “Symptomatic but Content” profiles (Clark & Malecki, 2022). Transgender identity is also strongly associated with poorer outcomes, as transgender students report higher levels of depression, insomnia, and overall psychological distress compared to their cisgender peers (Connolly et al., 2016). Age-related differences have also been identified, with younger individuals more likely to fall into “Troubled” profiles compared to older individuals (Xu et al., 2023). Finally, also romantic relationship status matters, as partnered individuals generally report better mental health and fewer psychological problems than single peers (Adamczyk & Segrin, 2015).

In the context of education, bachelor’s students reporting higher levels of depression, anxiety, and psychological distress compared to master’s and PhD students (Müller et al., 2022). Additionally, freshmen consistently show poorer mental health outcomes than students in later years (Janatolmakan et al., 2019). Finally, mental health disparities are also evident between domestic and international students, with international students reporting significantly higher levels of depressive symptoms, anxiety, and perceived stress compared to domestic peers (Amanvermez et al., 2024).

Beyond these demographic differences, insomnia is closely linked to both psychological distress and reduced well-being among university students (Carrión-Pantoja et al., 2022), and is increasingly recognized as a transdiagnostic indicator of mental health vulnerability (Gardani et al., 2022). Accordingly, examining insomnia severity across profiles provides an important external validation of their clinical relevance. If the identified profiles meaningfully capture variation in mental health functioning, they should also differ in sleep-related impairment.

## The Current Study

To address the limited understanding of mental health heterogeneity among help-avoidant university students, the present study examined mental health profiles in this group. Building on the dual-factor model of mental health, the identified profiles were expected to broadly reflect the four commonly described categories: Complete Mental Health, Symptomatic but Content, Vulnerable, and Troubled (Hypothesis 1). The study further examined whether mental health profile structure and distribution differed between help-avoidant students and students who currently or previously sought professional help, as help-seeking behavior may vary depending on symptom profile structure (Hypothesis 2). In addition, the study tested whether demographic characteristics were associated with profile membership and whether profiles differed in levels of insomnia severity, with higher psychopathology expected to be associated with poorer sleep (Hypothesis 3).

## Method

### Procedure and Participants

Data were drawn from Caring Universities, the Dutch branch of the World Health Organization’s World Mental Health International College Student (WMH-ICS) initiative. WMH-ICS is a large cross-national research collaboration embedded within the broader WHO World Mental Health Survey program. The WMH-ICS conducts standardized web-based surveys to university students to assess the prevalence, correlates, and functional impact of common mental disorders. The initiative also supports the development and evaluation of scalable, internet-based mental health interventions (Cuijpers et al., 2019). Ethical approval was granted by the Scientific and Ethical Review Board of Vrije Universiteit Amsterdam. Between November 2022 and January 2023, all students from the participating institutions were invited via email (*N* = 208,796) to participate in the survey, which was described as a voluntary scientific study examining student mental health to inform university support policies. The information letter outlined the study purpose, what participation involved, potential advantages and disadvantages. Students were informed that participation was voluntary and that they could withdraw at any time without explanation. Digital informed consent was obtained prior to participation. Responses were treated confidentially and pseudonymized, with identifying information stored separately in accordance with GDPR regulations. No financial compensation was provided. To enhance participation, reminder emails were sent to non-respondents. A total of 9055 (4.3%) students provided consent and completed the questionnaires. Little’s MCAR test indicated that data were missing completely at random (*p* > .05); and thus, 324 cases (3.6%) with missing values were removed. Mahalanobis distance was applied to detect outliers, with 52 cases identified as extreme values (*p* < .001) and excluded. The final sample comprised 8,679 participants (*M*_age_ = 21.76, *SD* = 3.31; 69.3% female).

### Measures

#### Help-Seeking Behavior

Help-avoidant students were identified using two screening questions: (1) “*Was there ever a time in the past 12 months when you felt that you might need psychological counseling*,* medication*,* or some other type of treatment for any emotional or substance use problems?*” and (2) “*Did you ever in your life receive psychological counseling*,* medication*,* or some other type of treatment for an emotional or substance use problem?*”. Students who answered “*Yes*” to the first question and “*No*” to the second were classified as Help-avoidant, yielding a subsample of 2,023 participants. For profile comparisons, the following additional groups were identified (a) Current help-seeking: students who responded “*Yes*” to both questions and reported receiving more than one month of psychological support in the past 12 months (*n* = 2,493), based on a follow-up item (“*About how many months in the past 12 months did you receive psychological counseling*,* medication*,* or some other type of treatment for an emotional or substance use problem?*”), and; (b) Past help-seeking: students who answered “*Yes*” to both questions but indicated to have received zero months of help in the past 12 months (*n* = 1,093), based on the same follow-up item.

#### Subjective Well-Being

Subjective well-being was assessed using the Mental Health Continuum–Short Form (MHC-SF; Keyes, 2002), which includes 14 items covering three dimensions of well-being: emotional well-being (EWB; 3 items), psychological well-being (PWB; 6 items), and social well-being (SWB; 5 items). Sample items include *“During the past month*,* how often did you feel”: “happy?*” (EWB), “*that your life has a sense of direction or meaning to it?*” (PWB), and “*that you belonged to a community?*” (SWB). Participants rated the frequency of their experiences over the past month on a 6-point scale ranging from 0 (*never*) to 5 (*every day*). Subscale scores were computed by summing the items within each subscale, with higher scores indicating greater EWB, PWB, and SWB. The MHC-SF exhibited acceptable internal consistency, with Cronbach’s α values of 0.86 for EWB, 0.84 for PWB, 0.72 for SWB, and 0.90 for the overall scale.

#### Psychopathology

The Irrational Procrastination Scale (IPS; Steel, 2010) was used to assess procrastination. The IPS consists of 9 items with a 5-point Likert scale (response categories range from 1 = *very seldom*,* or not true of me* to 5 = *very often true*,* or true of me*) assessing the degree of irrational delay causing procrastination. A sample item is “*I delay tasks beyond what is reasonable*”. Three items were reverse coded prior to computing the total score. A total score was calculated by summing all items, with higher total scores reflecting greater self-reported procrastination. The scale showed good internal consistency with Cronbach’s α values of 0.90.

Patient Health Questionnaire-9 (PHQ-9; Kroenke et al., 2001) is a 9-item self-report measure used to assess the severity of depressive symptoms. Respondents rate how often they experienced each symptom using a 4-point scale ranging from 0 (not at all) to 3 (nearly every day). A sample item is “*Over the last 2 weeks*,* how often have you been bothered by little interest or pleasure in doing things?*”. A total score is obtained by summing all items, with higher scores indicating greater severity of depression. The scale demonstrated good internal consistency with a Cronbach’s α of 0.84.

The 7-item Generalized Anxiety Disorder Scale (GAD-7; Spitzer et al., 2006) is a self-report questionnaire that assesses symptoms of general anxiety. Participants indicate how often they have been bothered by each symptom using a 4-point Likert scale ranging from 0 (not at all) to 3 (nearly every day). A sample item is “*Over the last 2 weeks*,* how often have you been bothered by feeling nervous*,* anxious*,* or on edge?*”. A total score is calculated by summing all items, with higher scores indicating greater levels of anxiety. Internal consistency for the GAD-7 was good with Cronbach’s α of 0.88.

The Perceived Stress Scale (PSS-10; Cohen, 1988) is a 10-item self-report questionnaire designed to measure the degree to which individuals perceive their lives as stressful. Items assess the frequency of feelings and thoughts related to stress, using a 5-point Likert scale ranging from 0 (never) to 4 (very often). A sample item is “*In the last month*,* how often have you been upset because of something that happened unexpectedly?*”. Higher total scores indicate greater perceived stress. Cronbach’s alpha for the PSS-10 was 0.79, indicating acceptable internal consistency.

#### Sociodemographic Variables

Sociodemographic information was collected via self-report. Assessed variables included age (in years), gender identity (male, female, other), and transgender identity (cisgender, transgender), relationship status (living together, steadily dating one person, dating one or more people but not in a steady relationship, not currently dating), academic level (bachelor, master), freshman status (freshman, non-freshman), and international background (international student, domestic student).

#### Insomnia

The Insomnia Severity Index (ISI; Bastien et al., 2001) is a 7-item self-report measure assessing the severity of insomnia symptoms over the past two weeks. Items evaluate difficulties with sleep onset, sleep maintenance, early morning awakening, satisfaction with sleep, interference with daily functioning, noticeability of impairment, and distress caused by sleep problems. Responses are rated on a 5-point Likert scale ranging from 0 (no problem) to 4 (very severe problem), with higher total scores indicating greater insomnia severity. A sample item is “*How satisfied are you with your current sleep pattern?*”. In the present study, Cronbach’s alpha for the ISI was 0.85, indicating good internal consistency.

### Statistical Analyses

Statistical analysis was conducted in SPSS 30 and Mplus 8.11. As the low intraclass correlations (range = 0.002 to 0.008) indicated minimal university-level variance, all analyses were conducted at the student level. Preliminary analyses were conducted before the main analyses to verify the appropriateness of subsequent steps and included: (1) descriptive statistics and bivariate correlations among all study variables, (2) a confirmatory factor analysis (CFA) to test whether the dual-factor model provided a better fit than a unidimensional structure across the full sample, and (3) Because establishing measurement invariance of latent profiles across subgroups is essential for valid group comparisons of emergent latent profiles (Olivera-Aguilar & Rikoon, 2018), measurement invariance across gender identity was tested within the help-avoidant group. Specifically, an unconstrained model, in which profile parameters were freely estimated across groups, was compared with a semi-constrained model that imposed equality constraints on the profile-defining parameters across gender groups. This comparison evaluates whether the latent profiles have the same structure and interpretation across groups, which is conceptually comparable to establishing metric invariance in factor models (Putnick & Bornstein, 2016). This decision was informed by prior evidence of sex-based differences in mental health profiles (Clark & Malecki, 2022).

Next, to examine the extent to which latent mental health profiles among help-avoidant students align with the dual-factor model (RQ1), a series of LPAs was conducted among help-avoidant students using dual-factor indicators of mental health. Model selection was guided by comparative fit indices, including the Akaike Information Criterion (AIC), Bayesian Information Criterion (BIC), and Sample-Size Adjusted BIC (SABIC), with lower values indicating better fit (Ferguson et al., 2020). The Lo-Mendell-Rubin Adjusted Likelihood Ratio Test (LMR) and the Bootstrap Likelihood Ratio Test (BLRT) were used to evaluate whether a model with k profiles provided a significantly better fit than a model with k – 1 profiles (Nylund et al., 2007). To assess whether the number, structure, or distribution of profiles differ across help-avoidant and comparison groups (RQ2), separate LPAs were conducted within current help-seeking and past help-seeking groups. The number, structure, and distribution of profiles were then descriptively compared across groups. Finally, to examine whether the identified profiles differed meaningfully in demographic characteristics and insomnia severity (RQ3), two sets of analyses were conducted among help-avoidant students. First, multinomial logistic regression analyses were conducted. Second, insomnia severity was examined as a distal outcome using the Bolck–Croon–Hagenaars (BCH) procedure, which accounts for classification error when comparing profile-specific means. This approach allowed for testing whether insomnia levels differed significantly across the identified profiles.

## Results

### Preliminary Analyses

#### Descriptive Statistics and Bivariate Correlations

Participants in the help-avoidant group were from seven Dutch universities: Leiden University (*n* = 337; 16.7%), Utrecht University (*n* = 353; 17.6%), Maastricht University (*n* = 281; 13.9%), Erasmus University Rotterdam (*n* = 167; 8.3%), and University of Amsterdam (*n* = 453; 22.4%), Vrije Universiteit Amsterdam (*n* = 309; 15.3%), Inholland University of Applied Sciences (*n* = 103; 5.1%). The distribution of students across universities in the other three comparison groups (current help-seeking and past help-seeking) was similar, indicating no substantial differences in institutional representation. The mean age of this group was 21.43 years (*SD* = 3.21). The majority identified as female (71.7%), and 2.6% reported a gender identity that did not align with their sex assigned at birth. Slightly more than half were single (53.2%). Most participants were bachelor’s students (69.1%), nearly half were freshmen (55.4%) and identified as being of Dutch origin (53.8%). Direct information on participants’ socioeconomic status was not collected in the present study. However, national statistics indicate substantial intergenerational continuity in educational attainment in the Netherlands. According to the OECD (2025), approximately 73% of young adults (aged 25–34) with at least one parent holding a tertiary degree attain tertiary education themselves, compared with 32% of those whose parents did not complete upper secondary education. Moreover, financial difficulties among Dutch university students appear relatively low (van Hooijdonk et al., 2023). This broader context is important when interpreting the findings of the current study, as Dutch university samples may disproportionately represent students from higher-educated families and may generally report relatively low levels of financial difficulty.

Using established cut-off criteria, 42.8% of participants scored ≥ 10 on the PHQ-9, indicating probable clinically significant depressive symptoms (Kroenke et al., 2001). Additionally, 41.8% scored ≥ 8 on the GAD-7, reflecting clinically meaningful anxiety symptoms (Plummer et al., 2016). More than half of the sample (54.1%) scored 20 or higher on the PSS-10, corresponding to high perceived stress levels that require therapeutic intervention (Sharif-Nia et al., 2024). Finally, 32.3% of participants scored ≥ 34 on the Irrational Procrastination Scale, placing them in the upper 25% of the overall sample distribution (rather than only the help-avoidant subgroup) and indicating relatively high levels of procrastination. Supplemental Table S1 presents descriptive statistics and bivariate correlations among all study variables. All indicators of mental well-being — emotional, social, and psychological well-being were positively correlated with each other (*p* < .01) and negatively associated with all psychopathology indicators (*p* < .01), including depression (PHQ), anxiety (GAD), perceived stress (PSS), and procrastination (IPS).

#### Factor Structure of Mental Well-Being and Psychopathology

The dual-factor model, conceptualizing well-being and psychopathology as distinct but related constructs, demonstrated superior fit to the unidimensional model in CFA when tested on the full sample across all four groups (see Supplementary Table S2). Model modification indices suggested that allowing the item *“Good at managing the responsibilities of your daily life”* (from the PWB subscale of the MHC-SF) to cross-load onto procrastination significantly improved model fit. Given that the dual-factor model conceptualizes well-being and psychopathology as distinct yet related dimensions (Suldo & Shaffer, 2008), this item was retained as a cross-loading in both models. This decision is consistent with prior evidence linking environmental mastery and procrastination through shared elements of planning, task management, and self-control (Ljubin-Golub et al., 2019). In both models, residual covariances were specified between items 5–11 of the MHC-SF, items 2–3 of the GAD, items 4–5 of the PSS, and items 3–4 of the PHQ. Although CFI and TLI values for the dual-factor model were slightly below the conventional cut-off of 0.90 (McDonald & Ho, 2002), previous work indicates that these indices may be underestimated in complex models (Shi et al., 2019), hence, the dual-factor model was retained for subsequent analyses.

#### Latent Profile Measurement Invariance Across Gender

Given the small sample size of students identifying as ‘other’ gender (*n* = 46), which is below the recommended threshold of *n* = 500, latent profile measurement invariance was tested only for male and female groups (Spurk et al., 2020). Supplementary Table S3 shows a three-profile solution emerged as the best-fitting model for both girls and boys within the help-avoidant group. To evaluate measurement invariance across gender identities, increasingly constrained nested models were compared (See Table S4). Comparing the unconstrained and semi-constrained models indicated improved model fit on BIC and SABIC when equality constraints were applied. Although the LRT was significant (*χ*² = 157.92 [*df* = 42], *p* < .001) and the AIC increased slightly, these indices are known to be overly sensitive to small differences which may be negligible from a practical perspective (Olivera-Aguilar & Rikoon, 2018). Therefore, based primarily on BIC and SABIC, measurement invariance across gender was considered acceptable.

### Main Analyses

#### Identifying Latent Mental Health Profiles Among Help-Avoidant Students

A series of LPAs were conducted within the help-avoidant group, all of which converged successfully (See Table 1). Entropy values were acceptable and showed minimal variation across models. Fit indices (AIC, BIC, SA-BIC) improved with additional profiles, but the gains diminished after the four-profile model. Moreover, LMRT was nonsignificant for the five-profile model, indicating that adding a fifth profile did not significantly improve fit. BLRT remained significant at the 6-profile solution; however, BLRT is known to be overly sensitive in very large sample, often detecting trivial improvements (Muthén, 2007). Therefore, taking both fit indices and theoretical interpretability into account, the four-profile solution was retained as the most optimal model.

**Fig. 2:**
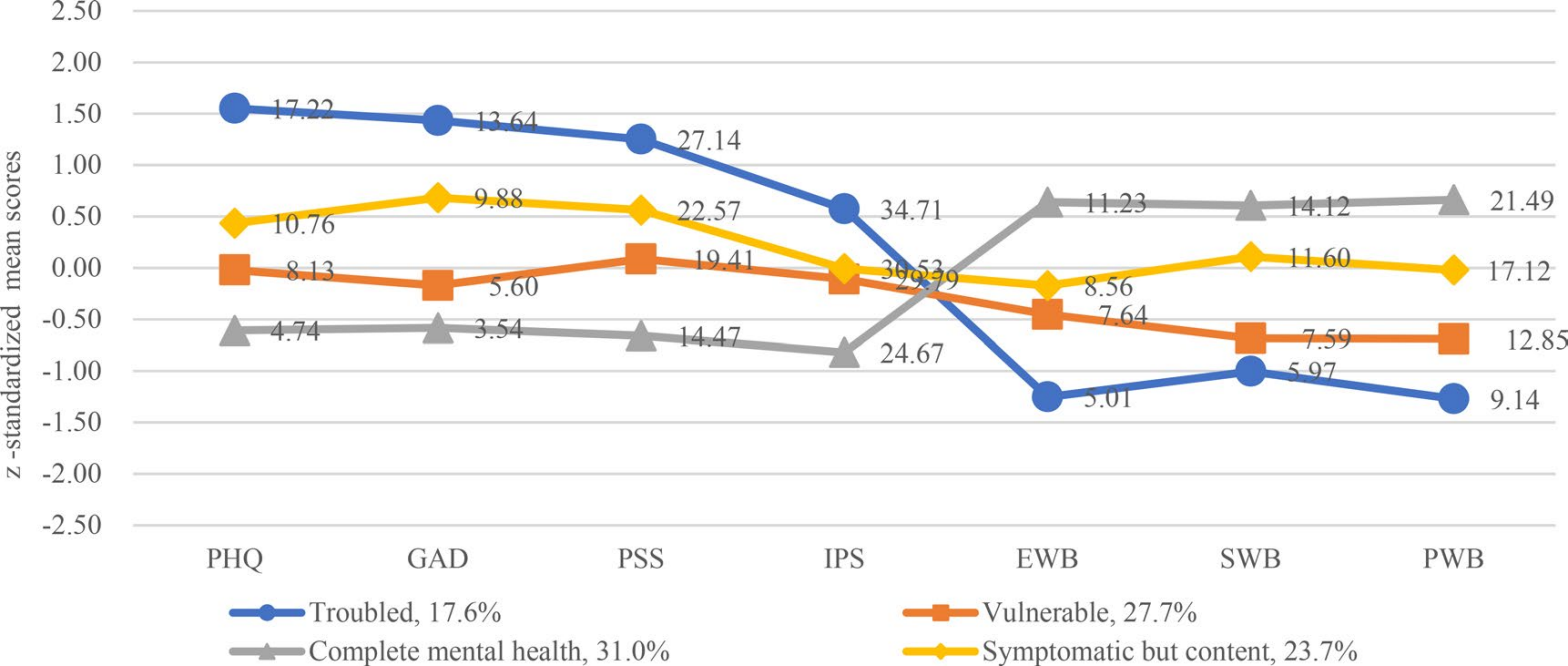
Z-Scores on Mental Health Indicators Across Profiles in the Help-Avoidant Group. Note: Z-standardized mean scores were calculated by subtracting the overall mean (across the help-avoidant, current help-seeking, and past help-seeking groups) from each group's mean for each indicator and dividing by the overall standard deviation; PHQ depression, GAD general anxiety, PSS perceived stress, IPS irrational procrastination, EWB emotional well-being, SWB social well-being, PWB psychological well-being; Data labels represent the unstandardized mean scores for each indicator within each profile.

Figure 2 shows the four-profile solution based on standardized (z-scored) mean scores across mental health indicators. The identified profiles were: (1) “Complete mental health” (31.0%, *n* = 628), with high levels of well-being and low levels of psychopathology; (2) “Symptomatic But Content” (23.7%, *n* = 479), with high-average psychopathology combined with relatively high well-being; (3) “Vulnerable” (27.7%, *n* = 560), with low-to-average well-being and moderately low psychopathology; and (4) “Troubled” (17.6%, *n* = 356), with low well-being and high psychopathology.

To further examine the significant differences in the means of the seven variables across profiles, a one-way MANOVA was used with all variables as outcomes and the profile membership as the grouping variable. Results showed significant associations of profile membership with all variables. Specifically, significant differences emerged for anxiety, *F*(3, 2019) = 1017.24, *p* < .001; depression, *F*(3, 2019) = 1219.35, *p* < .001; perceived stress, *F*(3, 2019) = 796.75, *p* < .001; procrastination, *F*(3, 2019) = 189.52, *p* < .001; EWB, *F*(3, 2019) = 779.48, *p* < .001; SWB, *F*(3, 2019) = 658.74, *p* < .001; and PWB, *F*(3, 2019) = 985.07, *p* < .001. The pattern of mean differences was consistent with the latent profile analysis results: students in the “Troubled” profile reported the highest levels of psychopathology and the lowest levels of well-being, whereas those in the “Complete Mental Health” profile showed the opposite pattern. The “Vulnerable” and “Symptomatic but Content” profiles showed intermediate patterns, with the “Symptomatic but Content” group reporting relatively high well-being despite elevated symptoms.

**Table 1:** Results of latent profile analysis for help-avoidant groups

Model	Profiles	Log likelihood	BIC	SA-BIC	AIC	LMR LRT *p*	BLRT *p*	Entropy	Profile prevalence
Planned	1	-43305.41	86717.39	86672.91	86638.82				1
	2	-41375.15	82917.76	82847.87	82794.29	< 0.001	< 0.001	0.81	0.46, 0.54
	3	-40647.58	81523.53	81428.22	81355.16	< 0.001	< 0.001	0.83	0.32, 0.19, 0.50
	**4**	**-40429.55**	**81148.36**	**81027.63**	**80935.09**	**< 0.05**	**< 0.001**	**0.77**	**0.18**,** 0.27**,** 0.31**,** 0.24**
Additional	5	-40228.46	80807.08	80660.94	80548.92	0.07	< 0.001	0.78	0.23, 0.27, 0.24, 0.19, 0.07
	6	-40082.82	80576.71	80405.14	80273.64	0.12	< 0.001	0.77	0.14, 0.22, 0.17, 0.23, 0.13, 0.10

Note. **Bold** indicates best fit; *BIC* Bayesian information criterion, *SABIC* sample-size adjusted bayesian information criterion, *AIC* akaike information criterion, *LMR* Lo-mendell-rubin likelihood ratio test, *BLRT* bootstrapped likelihood ratio

#### Group Difference in Latent Mental Health Profiles

**Fig. 3:**
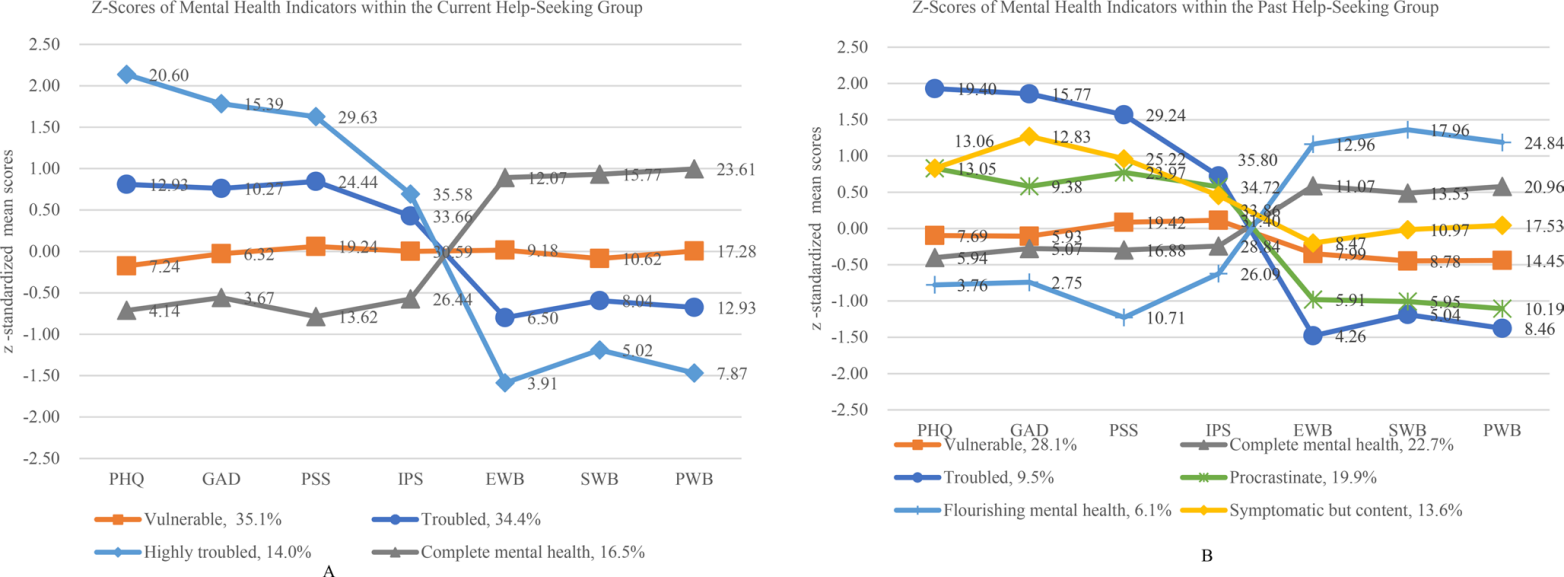
Z-Scores on Mental Health Indicators Across Profiles in the Current Help-Seeking and Past Help-Seeking Groups. Note: Z-standardized mean scores were calculated by subtracting the overall mean (across the help-avoidant, current help-seeking, and past help-seeking group) from each group's mean for each indicator and dividing by the overall standard deviation; PHQ depression, GAD general anxiety, PSS perceived stress, IPS irrational procrastination, EWB emotional well-being, SWB social well-being, PWB psychological well-being; Data labels represent the unstandardized mean scores for each indicator within each profile.

To examine whether profiles identified among help-avoidant students were distinct from other student groups, separate LPAs were conducted for the current help-seeking and past help-seeking groups. Supplementary Table S5 shows that a four-profile solution was optimal for the current help-seeking group, and a six-profile solution for the past help-seeking group (Ferguson et al., 2020). Based on Figs. 2 and 3, only the profiles in the help-avoidant group fully aligned with the dual-factor model. In contrast, among the current help-seeking group, four profiles were identified: “Vulnerable” (35.1%, *n* = 875), “Troubled” (34.4%, *n* = 858), and “Complete Mental Health” (16.5%, *n* = 412). Additionally, “Highly Troubled” (14.0%, *n* = 348) profile was identified, characterized by the poorest well-being and highest psychopathology, In the past help-seeking group, six profiles emerged: “Troubled” (9.5%, *n* = 104), “Vulnerable” (28.1%, *n* = 307), “Symptomatic but Content” (13.6%, *n* = 149), “Procrastinate” (19.9%, *n* = 218), marked by high procrastination relative to other symptoms, “Complete Mental Health” (22.7%, *n* = 248), and Flourishing (6.1%, *n* = 67), characterized by the highest well-being across all groups.

#### Demographic and Insomnia Differences Across Profiles

Supplementary Table S6 presents the demographic characteristics of each profile in the help-avoidant group. Age, gender identity, international background, academic level and freshman status significantly predicted profile membership (Supplementary Table S7).

Compared to the “Complete Mental Health” profile, students in the “Troubled” profile were more likely to be older, male, international, enrolled in a bachelor’s program, and in their first year of study. Specifically, older students were 1.06 times more likely to belong to the “Troubled” profile than younger students. Boys had 1.60 times greater odds of belonging to this profile than girls. International students had 2.12 times greater odds of membership compared to domestic students. Bachelor’s students were 2.12 times more likely than master’s students to be classified in this profile, and freshmen had 1.64 times greater odds of belonging to the “Troubled” profile compared to non-freshmen.

Students in the “Vulnerable” profile were more likely to be international, enrolled in a bachelor’s program, and freshmen. International students had 1.28 times greater odds of belonging to this profile relative to domestic students. Bachelor’s students were 1.55 times more likely than master’s students to be classified as “Vulnerable”, and freshmen were 1.43 times more likely than non-freshmen to belong to this profile.

Students in the “Symptomatic but Content” profile were more likely to be male, enrolled in a bachelor’s program, and freshmen. Boys had 1.95 times greater odds of belonging to this profile compared to girls. Bachelor’s students were 1.45 times more likely than master’s students to be classified in this profile, and freshmen had 1.31 times greater odds of membership compared to non-freshmen.

The BCH method was conducted to examine ISI scores as an outcome of latent profile membership. The result of the equality test of means across classes using the BCH procedure shows that insomnia severity significantly differs across the four profiles (*χ*^2^ = 444.74, *df* = 3, *p* < .001). Specifically, the “Troubled” profile exhibited the highest levels of insomnia (*M* = 20.78), which was significantly higher than all other groups (*p* < .001). Conversely, the lowest insomnia scores were found in “Complete Mental Health” (*M* = 13.92), while the “Symptomatic but Content” profile (*M* = 18.53) and “Vulnerable” profile (*M* = 15.34) showed moderate-to-high levels of insomnia. All pairwise comparisons between profiles were statistically significant (all *p* < .001).

## Discussion

Although previous research has shown that mental health status can influence students’ avoidance of professional help, the specific mental health profiles within this population remain poorly understood. This gap limits efforts to identify and support these students within educational settings. To address this issue, the current study applied a dual-factor model of mental health to examine latent profiles among university students reluctant to seeking professional help. Findings revealed that the profiles of help-avoidant students are consistent with the dual-factor framework and differed meaningfully from those of students reporting currently receiving help, and those with past help-seeking experience. In addition, demographic characteristics significantly predicted profile membership and insomnia severity increased progressively from the “Complete Mental Health” profile to the “Troubled” profile. The following sections further interpret these findings and discuss implications for research and practice.

Profiles as revealed within the help-avoidant group supported the dual-factor model, revealing four distinct profiles: “Troubled” (17.6%), “Vulnerable” (27.7%), “Complete Mental Health” (31.0%), and “Symptomatic but Content” (23.7%). Surprisingly, nearly one-third of students in this group had high well-being and low psychopathology despite acknowledging a perceived need for help. A likely explanation is that these students did not view their concerns as severe enough to warrant professional intervention, aligning with previous findings that low perceived severity is a common reason for not seeking services (Czyz et al., 2013). This finding suggests that one-third subset of help-avoidant students may not require additional intervention. However, students in the other three profiles may still benefit from psychological services. As avoidance can exacerbate mental health problems (Savage et al., 2016), there is a clear need for comprehensive assessment to identify help-avoidant students and implement effective intervention. Targeted efforts to reduce help-avoidant intentions, such as increasing awareness of symptoms, reducing self-stigma, and strengthening social connectedness, may help reduce persistent avoidance (Aguirre Velasco et al., 2020).

The comparison of profile membership across groups provides important insight into the nature of help avoidance. The distinction observed among help-avoidant students indicates that this group cannot be defined merely by experiencing less distress than help-seeking students. Rather, help avoidance may reflect distinct configurations of well-being and psychopathology. Notably, only the help-avoidant group demonstrated a profile structure that fully aligned with the dual-factor model, whereas the help-seeking groups lacked the “Symptomatic but Content” profile. The absence of this profile among current help-seeking students may suggest that individuals who maintain relatively high well-being despite elevated symptoms are less likely to perceive a need for professional support or may rely more on informal coping resources, such as peer support or self-management (Ryan et al., 2001). This finding suggests that well-being may play a role in suppressing help-seeking intentions, highlighting the importance of incorporating well-being indicators into screening and prevention efforts. Interestingly, the “Symptomatic but Content” profile also appeared in the past help-seeking group. This group included students who had sought professional help more than a year ago but either did not seek it again or received it for less than a month in the past year, despite perceiving a need. This pattern suggests that some past-help-seeking students may be subjectively reluctant to professional help, resembling the help-avoidant group. This resemblance may explain the presence of all four profiles in both groups. Moreover, the fact that profile membership among help-avoidant students fully aligned with the dual-factor model suggests that variability in well-being-symptom configurations is particularly pronounced within this group. This pattern further advances theoretical understanding of when the dual-factor framework is most informative, suggesting that it may be particularly well suited for examining mental health among help-avoidant students.

This study also examined demographic predictors of profile membership among help-avoidant students. Students in the “Troubled” profile were more likely to be older, male, international, enrolled in a bachelor’s program, and in their first year of study. Although older students were slightly more likely to belong to the “Troubled” profile, this difference was modest. This finding may be explained by the presence of older bachelor’s students (age > 30) in the “Troubled” profile, which raised its mean age (*M* = 21.27) slightly above that of the “Complete Mental Health” profile (*M* = 21.04). Older students are more likely to be on extended or non-traditional study paths, often due to academic difficulties or mental or physical health problems, which can elevate risk of poor mental health (Trenz et al., 2015). Furthermore, gender differences emerged. These findings aligns with previous research showing that males are more likely to fall into “Troubled” or at-risk profiles (Xu et al., 2023). This pattern may reflect gendered social norms around emotional expression, with males less likely to disclose common mental health problems due to social stigma, which then constrains help seeking (Tedstone Doherty & Kartalova-O’Doherty, 2010). In addition, men often report higher thresholds for acknowledging need, meaning that those who recognize difficulties and still avoid treatment may be more symptomatic (Yousaf et al., 2015). In contrast, females are generally more likely to seek informal support from peers or family members, which may contribute to better mental health outcomes (Azdi et al., 2025). International students, even from neighboring countries, often face challenges such as social adaptation, cultural and educational transitions, and academic adjustments (Rienties & Tempelaar, 2013). A Dutch study further reported that international students experienced higher levels of stress related to financial concerns and family health, which may, in turn, negatively affect their mental health (Amanvermez et al., 2024). The overrepresentation of bachelor’s and freshman students aligns with prior research indicating that early academic stages are associated with elevated psychological distress (Janatolmakan et al., 2019). Transitional periods involve substantial academic, social, and emotional adjustments, increasing vulnerability to poorer mental health (Sanagavarapu et al., 2019). In contrast, academically advanced students may experience lower stress levels as they have adapted to university demands (Puthran et al., 2016) and potentially developed more effective coping strategies (Ickes et al., 2015). Additionally, self-selection effects may contribute to these patterns, as students experiencing severe mental health difficulties earlier in their studies may discontinue their education and are therefore underrepresented in later academic stages (Zając et al., 2024). Moreover, mental health difficulties may indirectly impair academic performance, which then forces students to quit their studies (Chu et al., 2022). Conversely, poor grades themselves may exacerbate mental health problems, further increasing the likelihood of dropout (Hong et al., 2023).

Students in the “Vulnerable” profile were more likely to be international, enrolled in a bachelor’s program, and freshmen. This pattern suggests that students undergoing academic and cultural transitions may experience elevated psychological strain while not necessarily reaching the high levels of distress observed in the “Troubled” profile (Hirai et al., 2015). Unlike the “Troubled” group, the “Vulnerable” profile appears to reflect a more situational or transitional form of vulnerability rather than severe psychopathology. Specifically, students in this profile may be navigating adjustment-related challenges associated with entering university and, for international students, adapting to a new sociocultural and educational environment. Such stressors may temporarily impair well-being while not necessarily resulting in markedly elevated psychopathology compared to the “Troubled” profile. This distinction suggests that students in the “Vulnerable” profile may particularly benefit from early, transition-focused preventive support aimed at strengthening well-being and coping resources, rather than intensive clinical intervention.

Students in the “Symptomatic but Content” profile were more likely to be male, enrolled in a bachelor’s program, and freshmen. This pattern suggests that early-stage male students may report elevated symptoms while simultaneously maintaining relatively preserved levels of well-being. One possible explanation is that, despite the presence of distress, certain protective factors buffer the impact of stressors on overall well-being. Traits such as hardiness (Chuning et al., 2024) and supportive responses from significant others, including care, empathy, and emotional reassurance, can buffer the negative impact of stressors on well-being (Thoits, 2011). These buffering processes may help explain why students in this profile exhibit elevated psychopathology alongside relatively high levels of well-being.

Beyond these demographic patterns, insomnia severity differed significantly across the four help-avoidant profiles. Students in the “Troubled” profile exhibited the highest levels of insomnia, followed by those in the “Symptomatic but Content” profile, whereas the “Vulnerable” and “Complete Mental Health” profiles reported comparatively less sleep disturbances. These findings align with prior research demonstrating that insomnia is linked with psychological distress and reduced well-being among university students (Carrión-Pantoja et al., 2022) and further support its role as a transdiagnostic indicator of mental health vulnerability (Gardani et al., 2022). The observed pattern suggests that students in the “Troubled” profile experience pronounced sleep-related difficulties, reinforcing the external validity of the latent structure. Importantly, the “Symptomatic but Content” profile reported significantly higher insomnia levels than the “Vulnerable” and “Complete Mental Health” profiles, despite relatively high well-being. This pattern indicates that sleep impairment may be present even when subjective well-being remains intact, suggesting that professional support may also be warranted for students in this group. Overall, these findings underscore that among help-avoidant students—particularly those in the “Troubled” and “Symptomatic but Content” profiles—severe insomnia may signal substantial vulnerability despite reluctance to seek formal help.

### Implication for Research and Practice

The present study contributes to a fuller understand of mental health by applying the dual-factor model to evaluate mental health among help-avoidant university students. Notably, one-third of help-avoidant students exhibited complete mental health, underscoring the importance of distinguishing those who genuinely require support before implementing well-being interventions among help-avoidant students. Incorporating brief screening procedures prior to intervention may therefore enhance the precision and effectiveness of these programs. Moreover, profile membership, combined with demographic markers, may assist in identifying students unlikely to seek professional help despite being at elevated risk. By integrating such profile-based screening into student support services, universities may be better able to recognize help-avoidant students in need of support and provide timely, targeted interventions. Beyond screening, the distinct profiles highlight the importance of multitiered intervention approaches. Following Suldo and Shaffer (2008), all students could benefit from universal interventions (i.e., Tier 1) that promote well-being such as socio-emotional learning programs or peer support initiatives. Targeted, small-group interventions (Tier 2) may be especially useful for “Vulnerable” and “Symptomatic but Content” students, while students in the “Troubled” profile likely require more intensive, individualized services (Tier 3) to reduce psychopathology and improve well-being. Given that sleep problems are often more socially acceptable to disclose than emotional distress (Tucker et al., 2025), insomnia may serve as a gateway indicator of unmet mental health needs among help-avoidant students. Accordingly, interventions targeting sleep hygiene or insomnia symptoms may provide a less stigmatizing entry point for engaging this population in support services. Moreover, the emergence of a “Symptomatic but Content” profile also reinforces the importance of assessing both well-being and symptomatology, as elevated well-being can coexist with significant levels of psychopathology.

### Strengths, Limitations, and Future Directions

The present study has several strengths. First, the study draws on a large multi-university sample of students, which enhances the statistical power of the analyses and increases the robustness of findings. The large sample size also allowed for a more reliable identification of heterogeneous mental health patterns within the help-avoidant population. Second, this study applied the dual-factor model of mental health, which simultaneously considers both psychopathology and well-being, providing a more comprehensive conceptualization of students’ mental health. Third, the use of LPA enabled a person-centered examination of mental health heterogeneity among help-avoidant students, allowing for the identification of distinct mental health profiles that may not be detectable using variable-centered methods.

Despite these strengths, several limitations should be considered when interpreting the findings. First, the operationalization of help-seeking status represents a limitation. The survey questions used to distinguish current and past help-seeking relied primarily on whether students had accessed professional help within the current year versus earlier periods, which may not have been sufficiently robust to fully capture or differentiate mental health profile within the help-seeking groups. For example, some past help-seeking students (who sought help more than a year ago) should have been labelled as currently being help-avoidant, as they needed help but did not seek services in the past year. Because of structural barriers such as changes in academic or living circumstances, some students may have perhaps intentionally avoided to seek help recently. As a result, some misclassification between these groups cannot be ruled out. Alternative operationalizations of help-seeking status may yield somewhat different group distributions. Future research would benefit from more precise and comprehensive assessments of help-seeking behavior to better distinguish different forms of non-engagement with mental health services. Second, the cross-sectional design prevents determining whether profiles remain stable over time. Longitudinal research is needed to explore these profiles long-term, and to identify factors that may predict movement across profiles. For example, prolonged help avoidance may lead students to shift from “Complete Mental Health” to maladaptive profiles, with reduced well-being and worsened symptoms. Third, the study relied on self-report instruments. Although the effect of self-report bias is likely minimal in large samples (Fan et al., 2006), future studies still need to incorporate multi-information or clinician-rates assessment (e.g., peer-report or clinical diagnoses) for more comprehensive and robust evaluations.

## Conclusion

Although prior research has established the relationship between mental health and avoidance of professional help, the specific mental health profiles of help-avoidant university students had not been identified. The present study applied the dual-factor model which distinguishes between well-being and psychopathology, and identified four distinct profiles among help-avoidant students: Complete Mental Health, Vulnerable, Troubled, and Symptomatic but Content. These profiles differed meaningfully from those observed among current and past help-seeking students. Furthermore, older, male, bachelor’s, international, and freshman students were more likely to belong to non-healthy profiles, and insomnia severity increased progressively from the Complete Mental Health profile to the Troubled profile. These findings extend the dual-factor framework by demonstrating its applicability to help-avoidant populations and by revealing systematic differences in insomnia and demographic risk across profiles, with implications for the targeted identification and support of help-avoidant students in university mental health services.

## Electronic Supplementary Material

Below is the link to the electronic supplementary material.

Supplementary Tables

## Declarations

### Data Sharing Declaration

The datasets generated and analyzed during the current study are not publicly available but are available from the corresponding author on reasonable request.

### Preregistration

The study design, hypotheses, and planned analyses were preregistered on the Open Science Framework and can be accessed at: https://doi.org/10.17605/OSF.IO/Z2MTK
