# Supplementary material for: The Hidden Majority: Dual-Factor Mental Health Profiles Among Help-Avoidant University Students
Source: J Youth Adolesc. 2026 Jul 3;55(8):2208–23. doi: 10.1007/s10964-026-02380-3 (PMC13442322; doi:10.1007/s10964-026-02380-3)
Supplement: Supplementary file 1 [file 10964_2026_2380_MOESM1_ESM.docx]

**Table S1**

*Bivariate Correlation Matrix and Descriptive Statistics for Study Variables in the Total Study Population (N = 8679)*

| Variables | 1 | 2 | 3 | 4 | 5 | 6 | 7 |
| --- | --- | --- | --- | --- | --- | --- | --- |
| 1. EWB | 1 |  |  |  |  |  |  |
| 2. SWB | .63^**^ | 1 |  |  |  |  |  |
| 3. PWB | .73^**^ | .74^**^ | 1 |  |  |  |  |
| 4. PHQ | -.65^**^ | -.49^**^ | -.59^**^ | 1 |  |  |  |
| 5. GAD | -.54^**^ | -.41^**^ | -.48^**^ | .73^**^ | 1 |  |  |
| 6. PSS | -.62^**^ | -.47^**^ | -.56^**^ | .70^**^ | .72^**^ | 1 |  |
| 7. IPS | -.37^**^ | -.33^**^ | -.46^**^ | .50^**^ | .35^**^ | .48^**^ | 1 |
| Mean | 9.13 | 11.05 | 17.25 | 8.25 | 6.56 | 18.84 | 27.76 |
| Standard Deviation | 3.29 | 5.07 | 6.39 | 5.78 | 5.01 | 6.63 | 7.91 |
| Range | 0-15 | 0-25 | 0-30 | 0-27 | 0-21 | 0-40 | 9-45 |

*Note:* EWB = emotional well-being; SWB = social well-being; PWB = psychological well-being; PHQ = depressive symptoms; GAD = general anxiety; PSS = perceived stress; IPS = irrational procrastination; ^**^ *p* < . 01.

**Table S2**

*Summary of CFA Model Fit*

|  | Model chi-squared test | CFI | TLI | RMSEA | SRMR |
| --- | --- | --- | --- | --- | --- |
| Unidimensional | *χ^2^*(1115) = 35710.96, *p* < .001 | .85 | .85 | .06 | .06 |
| Dual factor | *χ^2^*(1114) = 27784.57, *p* < .001 | .89 | .88 | .05 | .05 |

*Note.* *χ²* = chi-squared test statistic; CFI = Comparative Fit Index; TLI = Tucker–Lewis Index; RMSEA = Root Mean Square Error of Approximation; SRMR = Standardized Root Mean Square Residual.

**Table S3**

*Preliminary Analysis of Multigroup LPA by Gender Identity among Help-Avoidant Students*

| Group | Number of profiles | | Log likelihood | BIC | SA-BIC | AIC | LMR LRT *p* | BLRT *p* | Entropy | Profile prevalence |
| --- | --- | --- | --- | --- | --- | --- | --- | --- | --- | --- |
| Girls (*N* = 1449) | 1 | -30991.90 | | 62085.69 | 62041.22 | 62011.79 |  |  |  | 1 |
|  | 2 | -29607.48 | | 59375.10 | 59305.21 | 59258.97 | < .001 | < .001 | .81 | .46, .54 |
|  | **3** | **-29053.66** | | **58325.68** | **58230.38** | **58167.32** | **< .001** | **< .001** | **.83** | **.52, .19, .29** |
|  | 4 | -29053.66 | | 58103.10 | 57982.38 | 57902.51 | .08 | .08 | .76 | .26, .29, .18, .27 |
| Boys (*N* = 528) | 1 | -11424.19 | | 22936.15 | 22891.71 | 22876.38 |  |  |  | 1 |
|  | 2 | -10880.61 | | 21899.13 | 21829.30 | 21805.21 | < .001 | < .001 | .85 | .54, .46 |
|  | **3** | **-10704.17** | | **21596.42** | **21501.19** | **21468.35** | **< .05** | **< .001** | **.83** | **.40, .44, .17** |
|  | 4 | -10637.35 | | 21512.92 | 21392.30 | 21350.70 | .22 | < .001 | .82 | .32, .13, .39, .15 |

*Note.* **Bold** indicates best fit; BIC = Bayesian Information Criterion; SABIC = Sample-Size Adjusted Bayesian Information Criterion; AIC = Akaike Information Criterion; LMR = Lo-Mendell-Rubin likelihood ratio test; BLRT = bootstrapped likelihood ratio test.

**Table S4**

*Measurement Invariance Across Gender Identity for 3-Profile Solution.*

|  | Log Likelihood | Scaling Correction Factor | Free Parameters | AIC | BIC | SABIC | Entropy |
| --- | --- | --- | --- | --- | --- | --- | --- |
| Unconstrained | -40725.76 | 1.27 | 89 | 81629.53 | 82126.98 | 81844.22 | 0.84 |
| Semi-Constrained | -40804.72 | 1.12 | 47 | 81703.45 | 81966.14 | 81816.82 | 0.84 |

*Note.* AIC = Akaike Information Criterion; BIC = Bayesian Information Criterion; SABIC = Sample-Size Adjusted Bayesian Information Criterion.

**Table S5**

*Results of Latent Profile Analysis for Help-Seeking (N =2492) and Past-Seeking (N =1093) Groups.*

| Group | Number of profiles | Log likelihood | BIC | SA-BIC | AIC | LMR LRT *p* | BLRT p | Entropy | Profile prevalence |
| --- | --- | --- | --- | --- | --- | --- | --- | --- | --- |
| Help-seeking | 1 | -54527.83 | 109165.15 | 109120.67 | 109083.65 |  |  |  | 1 |
|  | 2 | -51801.62 | 103775.30 | 103705.40 | 103647.24 | < .001 | < .001 | 0.84 | .51, .49 |
|  | 3 | -50838.57 | 101911.78 | 101816.47 | 101737.15 | < .001 | < .001 | 0.84 | .30, .48, .22 |
|  | **4** | **-50540.65** | **101378.52** | **101257.78** | **101157.31** | **< .001** | **< .001** | **0.80** | **.35, .34, .14, .17** |
|  | 5 | -50243.18 | 100846.13 | 100699.98 | 100578.35 | .10 | < .001 | 0.79 | .15, .28, .15, .25, .18 |
| Past-help-seeking | 1 | -23554.79 | 47207.53 | 47163.06 | 47137.57 |  |  |  | 1 |
|  | 2 | -22485.10 | 45124.13 | 45054.25 | 45014.20 | < .001 | < .001 | 0.82 | .48, .52 |
|  | 3 | -22113.55 | 44437.01 | 44341.72 | 44287.11 | < .001 | < .001 | 0.83 | .49, .27, .23 |
|  | 4 | -21980.33 | 44226.53 | 44105.83 | 44036.65 | < .01 | < .001 | 0.82 | .28, .20, .39, .13 |
|  | 5 | -21865.05 | 44051.95 | 43905.85 | 43822.10 | < .01 | < .001 | 0.79 | .22, .30, .11, .24, .14 |
|  | **6** | **-21798.23** | **43974.27** | **43802.75** | **43704.45** | **< .05** | **< .001** | **0.79** | **.28, .23, .10, .20, 06, .14** |
|  | 7 | -21745.91 | 43925.61 | 43728.68 | 43615.81 | .20 | < .001 | 0.78 | .18, .29, .12, .07, .09, .13, .12 |

*Note.* **Bold** indicates best fit; BIC = Bayesian Information Criterion; SABIC = Sample-Size Adjusted Bayesian Information Criterion; AIC = Akaike Information Criterion; LMR = Lo-Mendell-Rubin likelihood ratio test; BLRT = bootstrapped likelihood ratio test.

**Table S6**

*Demographic Composition of Mental Health Profiles among Help-Avoidant Students (N = 2023)*

|  | *N* (prevalence) or mean (standard deviation) | | | |  |
| --- | --- | --- | --- | --- | --- |
| Variable | Profile 1 | Profile 2 | Profile 3 | Profile 4 | Total (%) |
|  | Vulnerable  *N* = 560 (27.7%) | Complete mental health  *N* = 628 (31.0%) | Symptomatic but content  *N* = 479 (23.7%) | Troubled  *N* = 356 (17.6%) |  |
|  |  |  |  |  |  |
| Age | 21.27 (3.10) | 21.51 (2.95) | 21.06 (2.88) | 21.21 (3.00) |  |
| Gender identity |  |  |  |  |  |
| Male | 171 (32.4%) | 192 (36.4%) | 87 (16.5%) | 78 (14.8%) | 528 (100%) |
| Female | 376 (25.9%) | 422 (29.1%) | 388 (26.8%) | 263 (18.2%) | 1449 (100%) |
| Other | 13 (28.3%) | 14 (30.4%) | 4 (8.7%) | 15 (32.6%) | 46 (100%) |
| Transgender identity |  |  |  |  |  |
| Cisgender | 546 (27.7%) | 613 (31.1%) | 473 (24.0%) | 339 (17.2%) | 1971 (100%) |
| Transgender | 14 (26.9%) | 15 (28.8%) | 6 (11.5%) | 17 (32.7%) | 52 (100%) |
| Relationship |  |  |  |  |  |
| Married/engaged | 22 (23.2%) | 33 (34.7%) | 29 (30.5%) | 11 (11.6%) | 95 (100%) |
| Steady relationship | 171 (26.6%) | 229 (35.7%) | 149 (23.2%) | 93 (14.5%) | 642 (100%) |
| Unsteady relationship | 34 (17.6%) | 66 (34.2%) | 53 (27.5%) | 40 (20.7%) | 193 (100%) |
| Single | 333 (30.5%) | 300 (27.4%) | 248 (22.7%) | 212 (19.4%) | 1093 (100%) |
| Background |  |  |  |  |  |
| International | 265 (28.5%) | 260 (28.0%) | 195 (21.0%) | 209 (22.5%) | 929 (100%) |
| Domestic | 295 (27.0%) | 368 (33.6%) | 284 (26.0%) | 147 (13.4%) | 1094 (100%) |
| Freshman status |  |  |  |  |  |
| Freshman | 325 (28.9%) | 316 (28.1%) | 266 (23.7%) | 216 (19.2%) | 1123 (100%) |
| Non-freshman | 235 (26.1%) | 312 (34.7%) | 213 (23.7%) | 140 (15.6%) | 900 (100%) |
| Academic level |  |  |  |  |  |
| **Table S6** (continued) | | | | | |
| Bachelor | 396 (28.0%) | 407 (28.8%) | 348 (24.6%) | 263 (18.6%) | 1414 (100%) |
| Master | 164 (26.9%) | 221 (36.3%) | 131 (21.5%) | 93 (15.3%) | 609 (100%) |

*Note.* Steady relationship= steadily dating one person but not engaged; Unsteady relationship = dating one or more people but not in a steady relationship; Single = individuals not currently dating; Percentages represent the proportion of participants within each demographic category assigned to each latent profile.

**Table S7**

*Multinomial Logistic Regression Predicting Mental Health Profile Membership among Help-Avoidant Students (N = 2023)*

| Variable | Profiles | | | | | | | |
| --- | --- | --- | --- | --- | --- | --- | --- | --- |
|  | Complete Mental Health | | Troubled | | Vulnerable | | Symptomatic but Content | |
|  | OR | 95%CI | OR | 95%CI | OR | 95%CI | OR | 95%CI |
| Age | Reference | Reference | 1.06^*^ | [1.00, 1.12] | 1.03 | [0.98, 1.09] | 0.98 | [0.93, 1.04] |
| Male | Reference | Reference | Reference | Reference | Reference | Reference | Reference | Reference |
| Female | Reference | Reference | 0.63^**^ | [0.46, 0.86] | 0.97 | [0.76,1.25] | 0.51^***^ | [0.38, 0.69] |
| Cisgender | Reference | Reference | Reference | Reference | Reference | Reference | Reference | Reference |
| Transgender | Reference | Reference | 1.93 | [0.93, 4.03] | 1.00 | [0.47, 2.11] | 0.47 | [0.18, 1.24] |
| Married/engaged | Reference | Reference | Reference | Reference | Reference | Reference | Reference | Reference |
| Steady relationship | Reference | Reference | 1.25 | [0.60, 2.61] | 1.12 | [0.63, 1.99] | 0.65 | [0.38, 1.12] |
| Unsteady relationship | Reference | Reference | 1.79 | [0.80, 4.01] | 0.79 | [0.39, 1.54] | 0.81 | [0.43, 1.51] |
| Single | Reference | Reference | 1.99 | [0.95, 4.12] | 1.60 | [0.90, 2.83] | 0.78 | [0.46, 1.33] |
| Domestic students | Reference | Reference | Reference | Reference | Reference | Reference | Reference | Reference |
| International students | Reference | Reference | 2.12^***^ | [1.62, 2.78] | 1.28^*^ | [1.01, 1.62] | 0.99 | [0.77, 1.27] |
| Freshman -no | Reference | Reference | Reference | Reference | Reference | Reference | Reference | Reference |
| Freshman -yes | Reference | Reference | 1.64^**^ | [1.22, 2.19] | 1.43^**^ | [1.11, 1.84] | 1.31^*^ | [1.01, 1.10] |
| Master | Reference | Reference | Reference | Reference | Reference | Reference | Reference | Reference |
| Bachelor | Reference | Reference | 2.12^***^ | [1.45, 3.10] | 1.55^**^ | [1.12, 2.16] | 1.45^*^ | [1.02, 2.07] |

*Note.* Steady relationship= steadily dating one person but not engaged; Unsteady relationship = dating one or more people but not in a steady relationship; Single = individuals not currently dating; ^***^ *p* < .001, ^**^ *p* < .01, ^*^*p* < .05
